# Utilizing learning communities to deliver an integrated undergraduate medical education curriculum

**DOI:** 10.1080/10872981.2021.2011606

**Published:** 2021-12-12

**Authors:** Chi Braunreiter, Sathyanarayan Sudhanthar, Brad Riley, Kelly Armstrong, Brian Mavis, Jonathan Gold

**Affiliations:** a College of Human Medicine, Michigan State University, Grand Rapids, MI, USA; b Division of Pediatric Hematology and Oncology Spectrum Health Medical Group, Grand Rapids, MI, USA; c Office of Medical Education Research and Development, Michigan State University, Grand Rapids, MI, USA

**Keywords:** Learning communities, undergraduate medical education, curricular integration

## Abstract

**Aim:**

Learning communities (LCs) have been identified as a structure to support student wellness as well as create a positive learning environment and have been increasingly adopted in undergraduate medical education (UGME). In 2016, Michigan State University College of Human Medicine made curricular changes which integrated basic, social, and clinical sciences. One of the major strategies adopted to deliver this integrated curriculum was to create LCs that served as a central scaffold for students’ academic development. Our primary aim is to describe how the school utilized LC faculty to deliver this core integrated curriculum.

**Methods:**

Students were surveyed about their perceptions of the effectiveness of the LCs in delivering an integrated science curriculum. Student academic performance in the new curriculum was compared to that of students from the legacy curriculum as a measure of the effectiveness of the curricular changes.

**Results:**

The percentage of students in each class who responded to surveys ranged between 78.7% and 95.8%. Mean Likert responses (1 = strongly disagree; 5 = strongly agree) for statements ‘the Faculty Fellow is effective in helping me learn the scholar group content’, ‘the Faculty Fellow is an effective teacher in our scholar group’, and ‘the Faculty Fellow is well prepared for our scholar group’ ranged from 4.37 to 4.78, 4.72 to 4.76, and 4.81 to 4.86, respectively. In addition, a comparison of summative exam scores of the new curriculum’s students to the legacy curriculum’s students demonstrated comparable or better performances in the new curriculum.

**Conclusions:**

Utilizing LCs to deliver an integrated science curriculum is an underutilized strategy in UGME. Surveys on student satisfaction and academic performance are encouraging. Additional outcome measures are planned to continually evaluate this innovative multifaceted integration.

## Introduction

Undergraduate medical education (UGME) in the USA has traditionally involved 2 years of basic science training in the classroom setting followed by 2 years of clinical clerkships delivered in teaching hospitals [1,2]. Citing failures to learn, retain, and transfer basic science concepts to clinical patient care, critics of this segregated curriculum called for reform that would integrate the sciences [2–6]. Medical schools have utilized different instructional formats and strategies to integrate the sciences based on their own curricular needs and resources [2,7,8].

Along with calls for curriculum reform, there has been increasing interest in supporting medical student wellness. One approach to supporting wellness has been the development of learning communities (LCs) [9]. LCs are defined as an intentional and purposeful small group of students and faculty whose purpose is to create a positive learning environment by fostering long-term mentoring relationships, helping to form professional identities and a sense of well-being, and creating a sense of wholeness as a community [10–12].

Since its introduction in the USA medical education system in 1990 [13,14], periodic surveys of medical schools have shown increasing adoption of LCs [9,15,16]. Through an Association of American Medical Colleges (AAMC)-wide survey, Smith et al. reported in 2014 that 66 (*n* = 151) medical schools had an LC structure, and many more schools were considering starting one [9]. The Learning Communities Institute (LCI), a non-profit organization whose mission is to ‘promote, enhance, and evaluate’ LCs in medical education, has approximately 50 institutional and individual members [17]. A survey of medical schools that are members of the LCI reported that the most frequently cited objectives of LCs were to provide advising and mentoring, professional development, course offerings in clinical skills and medical humanities, social activities, and wellness [18]. LCs provide a supportive home to students outside the rigors of the necessary sciences [19].

LC faculty play a number of roles in students’ lives, including serving as role models, advising, mentoring, and coaching. The frequent contact between students and faculty in LCs has been shown to positively affect the learning environment [20]. Coaching in medicine is a longitudinal relationship between coach and student, providing ongoing feedback by direct observation of students in their learning process and assisting them in improving performance [21]. In an integrated curriculum, it is possible to have LC faculty play a central teaching role in basic sciences expanding their opportunities to observe and coach students directly.

Michigan State University College of Human Medicine (MSU-CHM) implemented a new curriculum integrating clinical, basic, and social sciences in 2016. As a part of the curricular change, LCs were created to not only provide social support, longitudinal mentoring and coaching but also to utilize the LC structure to play a central role in delivering integrated basic, clinical and social science content throughout the first 2 years of medical school. The purpose of this article is to describe our unique approach to the role of LC faculty and preliminary data about its effectiveness.

## Setting

MSU-CHM embarked on a new curriculum in Fall 2016, termed Shared Discovery Curriculum (SDC) (http://curriculum.chm.msu.edu/) [22]. The curriculum integrates basic and social sciences with clinical sciences organized by patient-centered complaints and concerns. To further support the learning environment of a rigorous integrated curriculum, MSU-CHM created Learning Societies, our version of LCs, that sit at the center of this curriculum.

Students are assigned to one of the four Learning Societies upon matriculation. They are further assigned to smaller scholar groups (SGs) within their Learning Societies. Each SG is composed of 7–9 students. The composition of each scholar group is determined by a ‘sorting hat’ process designed to ensure the diversity of each group by ethnic and cultural background, previous academic performance, and prior clinical experience.

Each SG is assigned a clinical faculty member who develops a longitudinal relationship with their students over the course of the first 2 years of medical school. In addition, a team of basic and social scientists complement the clinical faculty. The collection of faculty is known as Faculty Fellows. Faculty Fellows apply for the position and undergo an interview process. They serve as advisors, coaches, and mentors to the students in addition to teaching the core curricular content. To accomplish these goals, they are paid approximately 30% of a full-time salary. Together, the four Learning Societies and Faculty Fellows are known as the Academy. Organizationally, the Academy is distinct from departments such as Student Affairs or Academic Achievement.

Learning society SGs are the primary educational home for students. Students remain with their SG members for the first 2 years of medical school and attend learning activities together. Learning activities include large group sessions, anatomy and histology labs, patient simulation, and SG meetings. SG meetings occur for 2 hours, twice weekly in the first year and once weekly in the second year. These meetings serve two functions. First, they provide time and space for students to debrief their clinical experiences and make connections between real patient encounters and the curricular content. Second, students engage in active collaborative case-based learning. With the guidance of their Faculty Fellows, SGs learn, develop, and apply clinical reasoning skills while incorporating basic and social science knowledge into patient-centered clinical scenarios.

Students meet individually with their assigned SG clinical Faculty Fellows twice a semester for coaching and individualized learning planning. During these student-led meetings, their Faculty Fellows assist with identifying learning gaps, support the overall well-being of the student, provide mentorship for course selections, and advise on professional development. These all-inclusive coaching meetings set the stage for a longitudinal student–faculty relationship. Faculty Fellows also provide students with summative assessments of participation, professionalism, and attendance observed during SGs. These assessments are included in students’ portfolios which are used to inform competency-based assessment and promotion. Some Faculty Fellows are members of the Student Competence Committee. However, a Faculty Fellow is recused from discussions involving their own student.

Faculty Fellows convene weekly for faculty development sessions. Sessions support Fellows’ coaching roles and include topics such as how to deliver feedback, facilitating small group sessions, and identifying students in need of additional academic support. A portion of the sessions is dedicated to reviewing basic and social science content and how they relate to patient complaints and concerns. Sessions are directed toward educator development rather than becoming necessary science content experts.

## Methods

The curriculum was implemented in 2016 and matriculated five classes (Class of 2020, Class of 2021, Class of 2022, Class of 2023, and Class of 2024) at the time of this manuscript preparation. For ongoing curricular assessment and development, students were emailed surveys during the spring semesters of the first and second year of medical school that cover many curriculum-related topics, except for the Class of 2024 and the second year of Class of 2023 due to the COVID19 pandemic. Among these topics were survey statements to evaluate students’ perceptions of their SG clinical Faculty Fellows. One statement, ‘the Faculty Fellow is effective in helping me learn the scholar group content,’ was provided to all classes. Two statements, ‘the Faculty Fellow is an effective teacher in our scholar group’ and ‘the Faculty Fellow is well prepared for our scholar group,’ were added to the survey during Class of 2021’s second year of medical school. The survey responses were on a 5-point Likert scale (1 = strongly disagree, 2 = disagree, 3 = neutral, 4 = agree, and 5 = strongly agree). The mean of all responses was calculated for each of the three questions. Surveys were optional and anonymous. The Michigan State University Institutional Review Board waived the need for approval and the need to obtain consent for the analysis and publication of the anonymized, pre-existed data that was collected during normal ongoing curricular assessment.

We compared the new and legacy curriculum students’ academic performance on the Comprehensive Basic Science Examination (CBSE) at similar points in their medical school career. The CBSE tests course-specific components utilizing multiple-choice questions similar to Step 1 and Step 2 Clinical Knowledge USA Medical Licensing Examinations. Whereas the CBSE test was required during the first and second year of the SDC, it was optional for students in the legacy curriculum. The mean CBSE score was calculated for each class. Although CBSE exams were administered throughout the COVID-19 pandemic, these scores were not included in the analysis as the curriculum was modified to accommodate quarantine and social-distancing restrictions.

## Results

**Figure 1.**
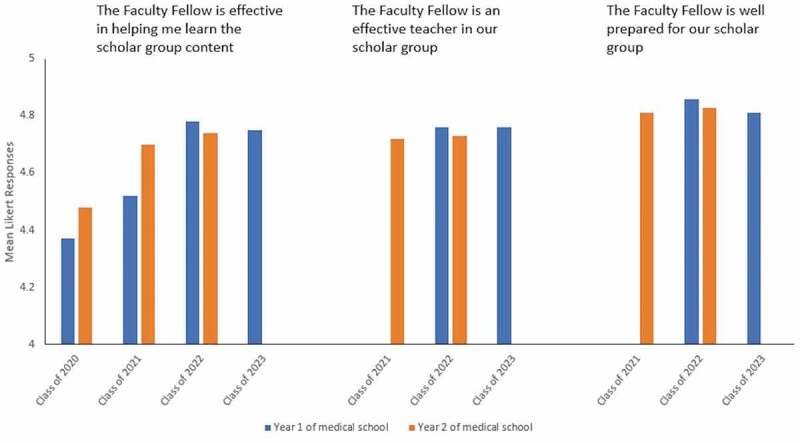
Student survey responses measuring the impact of the Faculty Fellow on student learning

The number of students in Classes 2020 to 2023 ranged from 181 to 201 students per year. The percentage of students in each class who responded to surveys ranged between 78.7% and 95.8% (Table 1). Mean Likert responses for the statement ‘the Faculty Fellow is effective in helping me learn the scholar group content’ ranged from 4.37 to 4.78 and gradually increased from the first year of implementation. Mean Likert responses for the statement ‘the Faculty Fellow is an effective teacher in our scholar group’ ranged from 4.72 to 4.76, while responses for the statement ‘the Faculty Fellow is well prepared for our scholar group’ ranged from 4.81 to 4.86 (Figure 1). Much like the rest of the world, the COVID-19 pandemic caused by the SARS-CoV-2 virus disrupted medical education at MSU-CHM in March 2020. For this reason, partial data were available for the Class of 2023 (disruption at the end of their first year), and no data is available for the Class of 2024 (disruption since the start of medical school).

**Table 1.** Survey completion rate by class

	Year 1 of medical school *n* of respondents/*n* of students in class (%)	Year 2 of medical school *n* of respondents/*n* of students in class (%)
Class of 2020	144/183 (78.7%)	136/181 (75.1%)
Class of 2021	154/191 (80.6%)	197/201 (98.0%)
Class of 2022	183/191 (95.8%)	160/192 (83.3%)
Class of 2023	173/188 (92.0%)	Not available

*n* = number.

**Figure 2.**
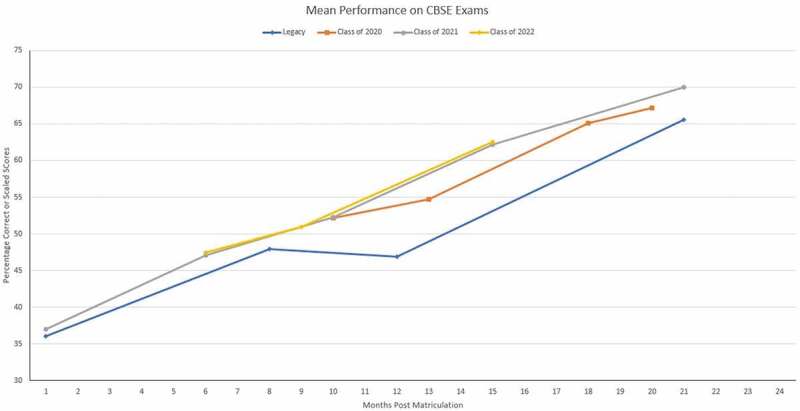
Mean Comprehensive Basic Science Examination (CBSE) scores of the SDC’s first three classes compared to the legacy curriculum students’ CBSE scores. The CBSE tests course-specific components utilizing multiple-choice questions similar to Step 1 and Step 2 Clinical Knowledge USA Medical Licensing Examinations. The scores on the standard CBSEs are scaled to first-time Step 1 test takers with a mean of 70 and a standard deviation of 8. Legacy students’ scores are percentage correct

Class of 2020 and 2021 students performed similarly to legacy curriculum students at comparable time points in the curriculum. The mean performance on CBSE exams for students in the legacy curriculum and Classes 2020, 2021, and 2022 are shown in Figure 2. The mean number of legacy curriculum students who voluntarily tested at approximately 1, 8, 12, and 21 months after matriculation was 200, 203, 201, and 177, respectively. This sample size is comparable to class sizes of Class of 2020 (*n* = 183), Class of 2021 (*n* = 191), and Class of 2022 (*n* = 191). At the time of this manuscript preparation, available data for Class of 2022 track similarly to Class of 2020 and 2021.

## Discussion

LCs have been shown to positively affect medical students’ performance in clerkships [23,24], improve faculty advising [25], and improve connectedness with faculty [26]. LCs, while used effectively in the undergraduate setting to promote active learning [12,18], are underutilized to deliver an integrated UGME curriculum in its entirety. Among the AAMC medical schools surveyed by Smith et al. in 2014 that identified as having LCs, only 14% reported delivering ‘PBL or case-based instruction’, while 26% reported delivering ‘other areas of the core curriculum’ using LCs [9]. Our experience demonstrates that LCs can be effective in delivering the core basic and clinical science components of an integrated curriculum in addition to providing coaching, mentoring, and social support. Students are major stakeholders in the LC structure, and persistent positive feedback indicates that they support this framework as well.

Like most LC faculty, our Faculty Fellows are a collection of clinician-teachers and academic scientists [27]. Student surveys were used to measure the impact of Faculty Fellows on learning. Initial scores for the first two classes demonstrated a high degree of satisfaction with having Fellows teach a broad swath of the curriculum (Figure 1). The subsequent improvement in satisfaction scores in the latter two classes may be secondary to ongoing faculty development sessions developed to promote excellence in teaching and coaching. Modifications and improvements in faculty development sessions were made during the first 2 years of the curriculum. These improvements included parceling out more time during faculty development sessions to review the more difficult basic science concepts and collaborating with educational specialists to improve teaching strategies in the small group setting. This collaboration led to a successful educator-directed professional development course, named Medical Educator Excellence in Teaching, whereby Faculty Fellows develop a teaching strategy based on observed gaps and implement the strategy in their small group sessions.

SDC students’ performance on CBSE appears to track similarly compared to the legacy curriculum students’ scores. It is difficult to determine if learning and retaining complex concepts of a fully integrated curriculum are enhanced solely by our Learning Societies. Data such as scores on benchmark exams or student satisfaction scores may reflect multiple changes with a major curriculum revision. Changes to our curriculum include the creation of patients’ chief complaints and concerns, enhanced simulation experience, and early introduction to clinical experiences. It is encouraging, though, that CBSE scores of students from the SDC curriculum track similarly, if not better, than scores obtained during the legacy curriculum.

There are several limitations to this study. First, there were many modifications in teaching methods with the shift to the MSU-CHM SDC, with Learning Societies being a single part. This makes it difficult to attribute success to any particular change. Secondly, students reported happiness with their Faculty Fellows and the curriculum does not equate to it being an effective educational method. Lastly, we have had to shift much of our teaching from in-person to online during the COVID pandemic. While SGs continued with collaborative case-based learning remotely while supporting each other through social distancing and quarantine restrictions, we do not yet have data that addresses the effectiveness of our curriculum during this time.

## Conclusions

In summary, MSU-CHM restructured its curriculum to center around Learning Societies, demonstrating its feasibility. These societies assist with delivering basic science and clinical science concepts while maintaining the published benefits of LCs on student satisfaction and social support. Utilizing LCs to deliver an integrated science curriculum is an underutilized strategy, with potential benefits in the coaching relationship between students and faculty. Surveys on student satisfaction and academic performance are encouraging. Schools that adopt integrated curricula might consider the use of LCs as one potential resource for curriculum delivery.

## References

[1] Flexner A. Medical education in the USA and Canada. A report to the Carnegie Foundation for the Advancement of Teaching. Bulletin No. 4. Boston, Mass: Updyke; 1910.

[2] Hopkins R, Pratt D, Bowen JL, et al. Integrating basic science without integrating basic scientists: reconsidering the place of individual teachers in curriculum reform. Acad Med. 2015;90(2):149–6. 25140528 10.1097/ACM.0000000000000437

[3] Ling Y, Swanson DB, Holtzman K, et al. Retention of basic science information by senior medical students. Acad Med. 2008;83(10 Suppl):S82–5. 18820508 10.1097/ACM.0b013e318183e2fc

[4] Pawlina W. Basic sciences in medical education: why? How? When? Where? Med Teach. 2009;31(9):787–789. 19811182 10.1080/01421590903183803

[5] Wilkerson L, Stevens CM, Krasne S. No content without context: integrating basic, clinical, and social sciences in a pre-clerkship curriculum. Med Teach. 2009;31(9):812–821. 19811186 10.1080/01421590903049806

[6] Irby DM, Cooke M, O’Brien BC. Calls for reform of medical education by the Carnegie Foundation for the Advancement of Teaching: 1910 and 2010. Acad Med. 2010;85(2):220–227. 20107346 10.1097/ACM.0b013e3181c88449

[7] Papa FJ, Harasym PH. Medical curriculum reform in North America, 1765 to the present: a cognitive science perspective. Acad Med. 1999;74(2):154–164. 10065057 10.1097/00001888-199902000-00015

[8] Instructional formats used in the curriculum, academic year 2019-2020. Available from: https://www.aamc.org/data-reports/curriculum-reports/interactive-data/instructional-formats-used-curriculum

[9] Smith S, Shochet R, Keeley M, et al. The growth of learning communities in undergraduate medical education. Acad Med. 2014;89(6):928–933. 24871245 10.1097/ACM.0000000000000239

[10] McMillan D, Chavis D. Sense of community: a definition and theory. J Community Psychol. 1986;14(1):6–23.

[11] Ebers L, Lenning O. The powerful potential of learning communities: improving education for future. Washington, DC. Graduate School of Education and Human Development, The George Washington University, ASHE-ERIC Higher Education Report, Vol 26, No. 6; 1999.

[12] Fink J, Inkelas K. A history of learning communities within American higher education. New Directions Student Serv. 2015;149:5–14.

[13] Cox MD. Introduction to faculty learning communities. New Directions Teach Learn. 2004;2004(97):5–23.

[14] Hafferty FW, Watson KV. The rise of learning communities in medical education: a socio-structural analysis. J Cancer Educ. 2007;22(1):6–9. 17570801 10.1007/BF03174367

[15] Ferguson KJ, Wolter EM, Yarbrough DB, et al. Defining and describing medical learning communities: results of a national survey. Acad Med. 2009;84(11):1549–1556. 19858814 10.1097/ACM.0b013e3181bf5183

[16] Association of American Medical Colleges. Number of medical schools organizing students into colleges or mentorship groups. Available from: https://www.aamc.org/initiatives/cir/425510/19a.html

[17] Learning Communities Insititute.

[18] Shochet R, Fleming A, Wagner J, et al. Defining learning communities in undergraduate medical education: a national study. J Med Educ Curric Dev. 2019;6:2382120519827911. 30937385 10.1177/2382120519827911 PMC6434432

[19] Vuk J, McKee S, Tariq S, et al. A better learning community: mixed-methods reveal medical student preferences with implications for learning community design and implementation. J Med Educ Curric Dev. 2021;8:23821205211014895. 34104783 10.1177/23821205211014895 PMC8150433

[20] Osterberg LG, Goldstein E, Hatem DS, et al. Back to the future: what learning communities offer to medical education. J Med Educ Curric Dev. 2016;3. DOI:10.4137/JMECD.S39420 PMC5736294 29349325

[21] Deiorio NM, Carney PA, Kahl LE, et al. Coaching: a new model for academic and career achievement. Med Educ Online. 2016;21:33480. 27914193 10.3402/meo.v21.33480 PMC5136126

[22] Wagner M, Sousa. Michigan State University College of Human Medicine. Acad Med. 2020;95(9S):S240–S4. 33626691 10.1097/ACM.0000000000003329

[23] Jackson MB, Keen M, Wenrich MD, et al. Impact of a pre-clinical clinical skills curriculum on student performance in third-year clerkships. J Gen Intern Med. 2009;24(8):929–933. 19521738 10.1007/s11606-009-1032-7 PMC2710476

[24] Stassen M. Student outcomes: the impact of varying living-learning community models. Res Higher Educ. 2003;44(5):581–613.

[25] Levine RB, Shochet RB, Cayea D, et al. Measuring medical students’ sense of community and satisfaction with a structured advising program. Int J Med Educ. 2011;2:125–132.

[26] Brandl K, Schneid SD, Smith S, et al. Small group activities within academic communities improve the connectedness of students and faculty. Med Teach. 2017;39(8):813–819. 28440094 10.1080/0142159X.2017.1317728

[27] Dewey CM, Friedland JA, Richards BF, et al. The emergence of academies of educational excellence: a survey of U.S. medical schools. Acad Med. 2005;80(4):358–365. 15793021 10.1097/00001888-200504000-00012

